# Sulfiredoxin-1 attenuates injury and inflammation in acute pancreatitis through the ROS/ER stress/Cathepsin B axis

**DOI:** 10.1038/s41419-021-03923-1

**Published:** 2021-06-17

**Authors:** Jun He, Miaomiao Ma, Daming Li, Kunpeng Wang, Qiuguo Wang, Qiuguo Li, Hongye He, Yan Zhou, Qinglong Li, Xuyang Hou, Leping Yang

**Affiliations:** 1 Department of General Surgery, The Second Xiangya Hospital, Central South University, Changsha, Hunan 410011 China; 2 Department of Rehabilitation, The First People’s Hospital of Huaihua, University of South China, Hengyang, Hunan China; 3 Department of Laboratory Medicine, Second Xiangya Hospital, Central South University, Changsha, 410011 Hunan China; 4 Department of General Surgery, Taizhou Central Hospital, Taizhou University Hospital, Taizhou, Zhejiang 318000 China; 5 Department of General Surgery, Hunan Chest Hospital, Changsha, 410006 Hunan China

**Keywords:** Cell death, Pancreatic disease

## Abstract

Acinar cell injury and the inflammatory response are critical bioprocesses of acute pancreatitis (AP). We investigated the role and underlying mechanism of sulfiredoxin-1 (Srxn1) in AP. Mild AP was induced by intraperitoneal injection of cerulein and severe AP was induced by partial duct ligation with cerulein stimulation or intraperitoneal injection of L-arginine in mice. Acinar cells, neutrophils, and macrophages were isolated. The pancreas was analyzed by histology, immunochemistry staining, and TUNEL assays, and the expression of certain proteins and RNAs, cytokine levels, trypsin activity, and reactive oxygen species (ROS) levels were determined. Srxn1 was inhibited by J14 or silenced by siRNA, and overexpression was introduced by a lentiviral vector. Transcriptomic analysis was used to explore the mechanism of Srxn1-mediated effects. We also evaluated the effect of adeno-associated virus (AAV)-mediated overexpression of Srxn1 by intraductal administration and the protection of AP. We found that Srxn1 expression was upregulated in mild AP but decreased in severe AP. Inhibition of Srxn1 increased ROS, histological score, the release of trypsin, and inflammatory responses in mice. Inhibition of Srxn1 expression promoted the production of ROS and induced apoptosis, while overexpression of Srxn1 led to the opposite results in acinar cells. Furthermore, inhibition of Srxn1 expression promoted the inflammatory response by accumulating and activating M1 phenotype macrophages and neutrophils in AP. Mechanistically, ROS-induced ER stress and activation of Cathepsin B, which converts trypsinogen to trypsin, were responsible for the Srxn1 inhibition-mediated effects on AP. Importantly, we demonstrated that AAV-mediated overexpression of Srxn1 attenuated AP in mice. Taken together, these results showed that Srxn1 is a protective target for AP by attenuating acinar injury and inflammation through the ROS/ER stress/Cathepsin B axis.

## Introduction

The incidence of acute pancreatitis (AP) is increasing worldwide [1]. The overall case-fatality rate for AP is approximately 5% and is dramatically increased when severe pancreatitis develops [2, 3]. AP is initiated by premature activation of pancreatic proteases, followed by self-digestion of the pancreas and inflammatory response [4]. Although AP, especially severe AP, is still a deadly disease, there is no approved therapy for it. Elucidation to the molecular mechanisms of the injured pancreas and the interplay with the inflammatory system will help identify potential therapies.

Acinar cell injury is central to AP. Reactive oxygen species (ROS) are substantially elevated, paralleling a defect in antioxidant defense in experimental AP models and patients [5, 6]. Oxidative stress persistently occurs from disease onset to the recovery of clinical manifestations, characterized by reduced glutathione, elevated MDA levels, and reduced SOD activity in the pancreas [7–9]. In addition to acting as damaging agents, ROS are also signal-transducing molecules that trigger proinflammatory cytokine production and induce apoptosis in AP [10]. Sulfiredoxin-1 (Srnx1), an endogenous antioxidant protein, belongs to the sulfiredoxin family and plays an essential role in various bioprocesses [11–13]. Previous works have shown that Srxn1 promotes carcinogenesis and protects against ischemia/reperfusion injury and apoptosis in cardiac progenitor cells [12, 13]. Nevertheless, the role and underlying mechanisms of Srxn1 in AP are unclear.

Endoplasmic reticulum (ER) stress is mediated by the unfolded protein response (UPR) [10]. ER stress is a common reaction stimulated by pancreatic toxins. For optimization of disulfide bond formation, the ER needs an oxidizing environment, but the supraphysiological ROS from the extra ER easily disrupt the redox balance, resulting in ER stress [14, 15]. ER stress induces apoptosis when its protective mechanism is defective. Knockout of ATF6 expression attenuated acinar cell apoptosis by inhibiting ER stress [16]. In addition, PERK-eIF2α and ATF6-mediated UPR could activate NF-κB, a key transcription factor for proinflammatory pathways [17]. Thus, ER stress is a central bioprocess for the induction of apoptosis and activation of inflammation. However, the detailed mechanism of ER stress induction in acinar cells and how it influences the activation of inflammatory cells remains largely unknown. Furthermore, whether ER stress actively regulates the activation of trypsinogen in AP is still controversial.

In the present study, we established a mild AP model and two severe AP models to analyze the expression of SRXN1 and found that SRXN1 expression was significantly upregulated in mild AP but depressed in severe AP. Inhibition of SRXN1 expression by pharmacological or genetic methods led to markedly improved ROS levels, the elevated release of trypsin, induction of apoptosis, and activation of macrophages and neutrophils in vivo and in vitro. Rescuing the expression of SRXN1 decreased ROS levels and apoptosis induced by cerulein in acinar cells. Regarding the mechanism, inhibition of SRXN1 strongly activated ER stress via ROS induction and activation of Cathepsin B, which converts trypsinogen to trypsin, and inhibiting ER stress largely abrogated these effects in acinar cells. Furthermore, we demonstrated that adeno-associated virus (AAV)-mediated overexpression of Srxn1 by intraductal administration ameliorated the injury and inflammation in cerulein-induced AP in mice.

## Results

### SRXN1 expression is upregulated in mild AP but reduced in severe AP

**Fig. 1:**
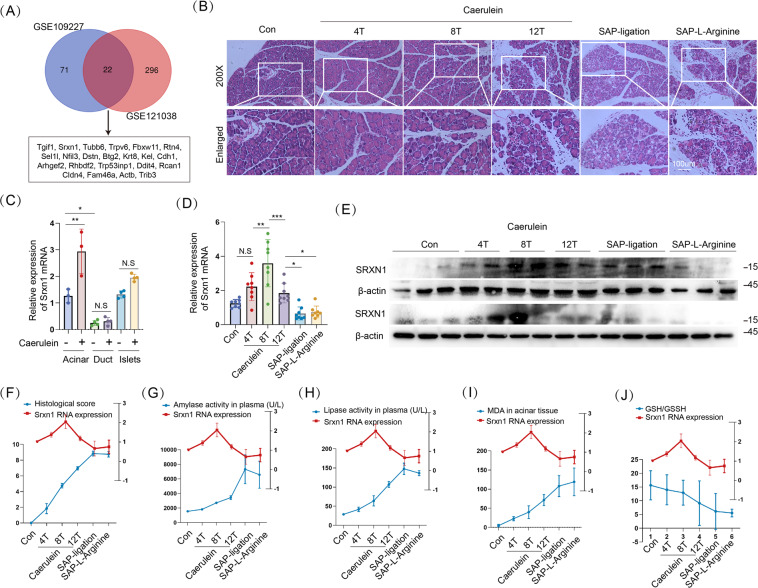
SRXN1 expression is upregulated in mild AP but reduced in severe AP. **A** In total, 22 genes commonly altered in both datasets by a cutoff of *p* < 0.05 and log_2_ fold change >0.5 or <−0.5. **B** H&E staining of pancreas in the control and the groups with cerulein-induced AP (4-, 8-, and 12-hourly injections), partial pancreatic duct ligation followed by injection of cerulein (50 µg/kg body weight) 2 days after surgery, and 3-hourly intraperitoneal injections of 3.3 g/kg L-arginine. **C** The expression of Srxn1 RNA in acini, ducts, and islets in the cerulein-induced AP and control mice. **D**, **E** The expression of Srxn1 RNA and protein in the cerulein-induced AP and the two SAP groups. **F** The histological score of the SAP groups was higher than that of the cerulein-induced AP group. **G**, **H** The trypsin activity of SAP was higher than that of the cerulein-induced AP group. **I** The MDA of pancreatic tissue in SAP was higher than that of the cerulein-induced AP group. **J** The GSH/GSSH ratio of pancreatic tissue in SAP was lower than that of the cerulein-induced AP groups. Con control; SAP severe acute pancreatitis; 4 T, 4-hourly injections; 8 T, 8-hourly injections; 12 T, 12-hourly injections; *n* = 8; *p < 0.05, **p < 0.01, ***p < 0.001.

To survey the transcriptomic profile of AP, we queried two GEO datasets, GSE109227 and GSE121038, which both compared the mRNA expression between cerulein-induced AP and controls, and identified 22 concomitantly altered genes (Fig. 1A). Among them, three genes, Srxn1, Sel1l, and Btg2, were selected for further research. The mouse model of mild AP is induced by intraperitoneal injection of cerulein, and SAP is induced by two methods: partial duct ligation followed by cerulein stimulation (Figure S1A) and intraperitoneal injection of L-arginine. Histological analysis demonstrated that these models were successfully induced (Fig. 1B). Sel1l expression was downregulated in mild and severe AP, and Btg2 expression was upregulated in pancreatitis, but the fold changes were less than 2 (Fig. S1B, C). Then, Srxn1 attracted our interest. Srxn1 mRNA was upregulated in acini, but not in pancreatic ducts and islets after cerulein stimulation (Fig. 1C). An interesting phenomenon was observed that Srxn1 was significantly upregulated in cerulein-induced AP (4-hourly and 8-hourly injection) but attenuated in the model with 12-hourly injections and further downregulated in the two SAP models (Fig. 1D, E). Histological score and amylase and lipase activities in plasma were used to evaluate the severity of AP. The expression of Srxn1 intersected with the histological score, amylase, and lipase activity with the increased severity of AP (Fig. 1F−H). Srxn1 is an endogenous antioxidant gene. Expectedly, the expression of Srxn1 also intersected with the MDA levels and GSH/GSSH ratios in pancreatic tissue from AP models (Fig. 1I, J). Thus, these data clearly indicated that Srxn1 expression was upregulated in mild AP but decreased in severe AP, suggesting its potential role in modulating the severity of AP in the mouse model.

### Inhibition of SRNX1 expression worsens AP in vitro and in vivo

**Fig. 2:**
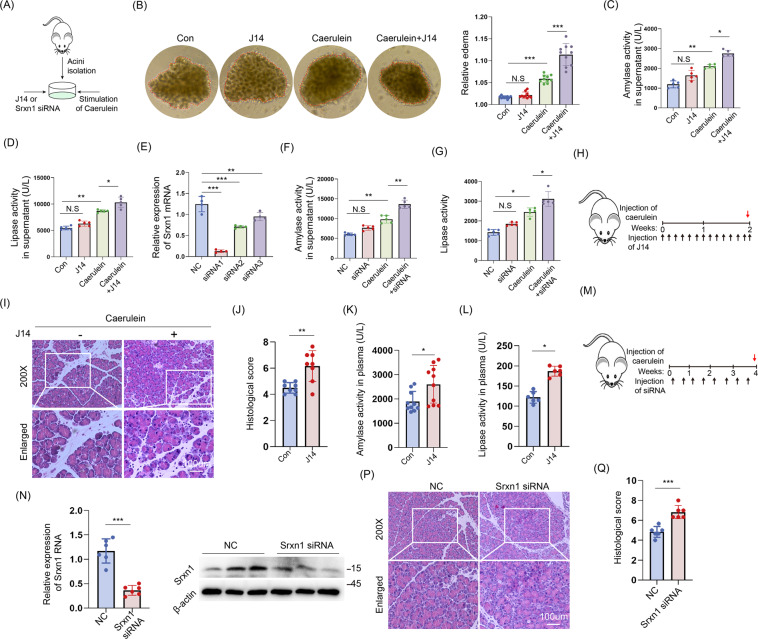
Inhibition of SRNX1 worsens AP in vitro and in vivo. **A** Schematic of the in vitro assay using isolated acini. **B** Cerulein (10 nM) for 10 min significantly promoted acini’s edema, and pretreatment with J14 (5 μM) for 6 h further promoted it. **C**, **D** Pretreatment with J14 (5 μM) for 6 h significantly increased trypsin activity induced by cerulein (100 nM, 1 h) in acini. **E** siRNA1 exhibited the highest efficiency and was used in the subsequent assays. **F**, **G** The trypsin activity of the siRNA+cerulein group was significantly higher than that of the cerulein group in acini. **H** J14 (in 10% DMAC + 10% Tween 80 + 80% saline) was intraperitoneally administered at a dose of 50 mg/kg body weight once a day for 2 weeks, and cerulein (50 µg/kg body weight) was administered by 8-hourly injection on the 13th day. **I**, **J** J14 strongly increased the histological score of cerulein-induced AP. **K**, **L** J14 significantly increased the trypsin activity of cerulein-induced AP. **M** Srxn1 siRNA was chemically modified and suitable for in vivo application. siRNA was administered by caudal vein injection at a dose of 20 nmol/mouse two times per week for a total of 4 weeks, and cerulein was injected 2 days after the last siRNA injection. **N** siRNA knockdown of the RNA and protein expression of Srxn1 in pancreatic tissue. **P**, **Q** Srxn1 siRNA increased the histology score of the pancreas induced by cerulein. Con control; NC negative control; N. S no significance, *p < 0.05, **p < 0.01, ***p < 0.001; data represent five or more experiments for in vitro assays and eight or more for in vivo assays.

Pancreatic acini were isolated and cultured as described previously [18] and are shown in Fig. 2A. J14 is a specific inhibitor of SRXN1, and its ability to eliminate ROS was verified in acinar cells (Figure S2A). Stimulation with cerulein (10 nM) led to obvious edema in vitro, and inhibition of SRXN1 expression by J14 (5 μM) further augmented the severity of edema (Fig. 2B). Amylase and lipase activities in the supernatant were also increased in the J14+cerulein group compared to the cerulein group (Fig. 2C, D). Furthermore, knockdown of Srxn1 expression significantly increased amylase and lipase activities in the supernatant compared to those of the control when stimulated by cerulein (Fig. 2E–G). To verify these effects in vivo, we administered J14 by intraperitoneal injection for 2 weeks and then stimulated the mice with cerulein by 8-hourly injection (Fig. 2H). J14 significantly increased the histological score (Fig. 2I, J) and amylase and lipase activities in plasma compared to those in the cerulein group (Fig. 2K, L). Furthermore, chemically modified Srxn1 siRNA, which is suitable for in vivo application, was administered by caudal vein injection two times per week for 4 weeks (Fig. 2M). Resembling the effects of J14, Srxn1 siRNA led to improvements in the histological score and trypsin activities in plasma upon cerulein (Fig. 2N–Q and Fig. S2B). These results suggested that inhibition of SRXN1 worsened the severity of AP in vitro and in vivo.

### SRXN1 attenuates AP by regulating oxidative stress and apoptosis

**Fig. 3:**
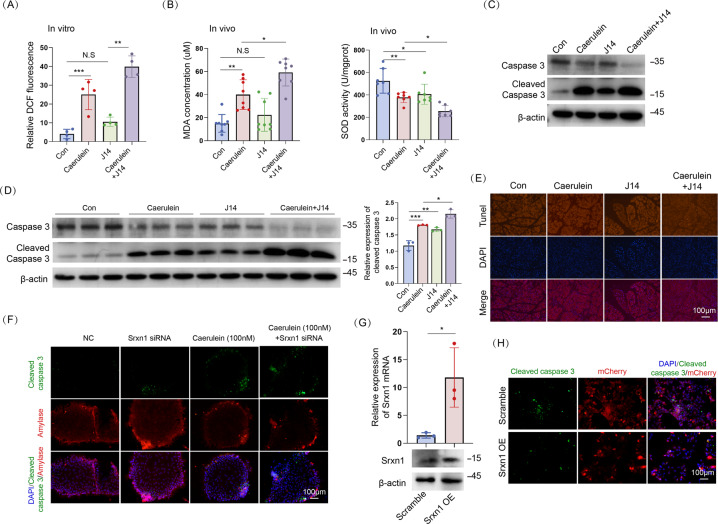
SRXN1 attenuates AP by inducing oxidative stress and apoptosis. **A** J14 (5 μM) significantly increased the DCF signal induced by cerulein (100 nM) in isolated acini. **B** J14 increased the MDA concentration and decreased SOD activity upon cerulein treatment in mice. **C** The cleaved caspase 3 level was substantially induced by cerulein (100 nM), and cotreatment with J14 further augmented it in acinar cells. **D**, **E** The cleaved caspase 3 was induced by cerulein at a dose of 50 µg/kg body weight eight times, and injection of J14 further promoted it in mice; TUNEL assay showed the same trend. **F** Acinar cells were isolated and cultured for 3 days, and Srxn1 siRNA was transfected for another 3 days and then cerulein (100 nM) was added. Amylase served as an indicator of acinar cells. **G**, **H** Lentivirus vector (mCherry) was transfected to overexpress Srxn1; IF staining of cleaved caspase 3 showed that Srxn1 overexpression prevented apoptosis induced by cerulein in acini. N. S no significance; Con control; NC negative control; OE overexpression; *p < 0.05, **p < 0.01, ***p < 0.001. Data represent three or more experiments for in vitro assays and eight for in vivo assays.

As the protective role of SRXN1 on AP was firmly demonstrated, we further analyzed its potential mechanism. DCFH can be oxidized by ROS to DCF, which emits fluorescence and is positively associated with the level of ROS. Expectedly, J14 increased the DCF signal compared to that of the control group and significantly increased it upon stimulation with cerulein in isolated acini (Fig. 3A and Fig. S3A). Srxn1 siRNA also increased the DCF signal upon treatment with the same concentration of cerulein (100 nM) (Figure S3B). Accordingly, J14 led to a strongly enhanced level of MDA and decreased level of SOD (Fig. 3B), both of which showed that inhibition of SRXN1 improved ROS levels in vivo. A TUNEL assay was used to detect apoptosis in pancreatic tissues. Administration of J14 led to low levels of apoptosis but induced pronounced apoptosis upon stimulation with cerulein (Fig. 3C). Western blots and immunofluorescence staining of cleaved caspase 3 showed the same trend: J14 significantly augmented the proapoptotic effect of cerulein in vivo and in vitro (Fig. 3D, E, and Fig. S3C). Furthermore, colabeling of amylase, which is exclusively expressed by acinar cells, and cleaved caspase 3, suggested that Srxn1 siRNA facilitated apoptosis in vitro in response to cerulein (Fig. 3F). The lentivirus vector Srxn1 was transfected into acinar cells to overexpress SRXN1. The lentivirus vector Srxn1 successfully augmented Srxn1 at the RNA and protein levels in acinar cells (Fig. 3G). Overexpression of Srxn1 obviously attenuated the expression of cleaved caspase 3 induced by cerulein (Fig. 3H). Therefore, these results showed that Srxn1 could alleviate AP by modulating ROS and apoptosis in vitro and in vivo.

### Inhibition of Srxn1 expression results in activation of M1 phenotype macrophages and neutrophils

**Fig. 4:**
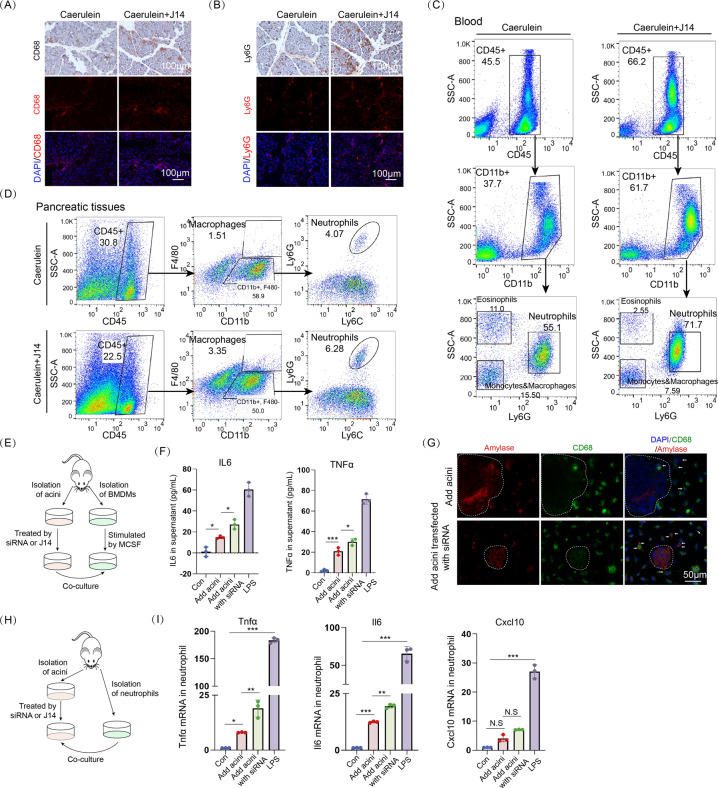
Inhibition of Srxn1 results in accumulation and activation of M1 macrophages and neutrophils. **A**, **B** Infiltrating M1 phenotype macrophages and neutrophils were stained with CD68 and Ly6g, respectively, in pancreatic tissues. **C** Flow cytometry analysis of blood showed an increase of neutrophils (CD45^+^/CD11b^+^/Ly6G^+^) in J14 + cerulein group. **D** Flow cytometry analysis of pancreatic tissues showed an increase of infiltrating neutrophils (CD45^+^/CD11b^+^/Ly6C^+^/Ly6G^+^) and macrophages (CD45^+^/CD11b^+^/F4/80^+^) in J14 + cerulein group. **E** BMDMs were isolated from marrow and stimulated for 7 days with MCSF (20 ng/mL) to differentiate into M1 phenotype macrophages and then cocultured with acinar cells. **F** Srxn1 siRNA largely increased the protein expression of IL6 and TNFα upon stimulation with cerulein compared with the control. LPS (500 ng/mL) served as a positive reference. **G** IF staining of CD68 and amylase showed that macrophages phagocytized more trypsin released from Srxn1-knockdown acini than normal acini. The white circle indicates acinar cells. **H** Schematic of coculture between acinar cells and neutrophils. **I** Neutrophils cocultured with acini transfected with Srxn1 siRNA showed significantly elevated RNA expression of Tnfα, Il6, and Cxcl10 compared with those cocultured with normal acini. LPS (500 ng/mL) served as a positive reference. Con control; *p < 0.05, **p < 0.01, ***p < 0.001. Data are three or more experiments for in vitro assays and eight for in vivo assays.

Then, we evaluated whether inhibition of Srxn1 expression influences the accumulation and activation of M1 phenotype macrophages and neutrophils, the dominant leukocyte populations infiltrating the pancreas during AP. Coadministration of cerulein and J14 increased the population of CD68 (M1 phenotype macrophages) or Ly6G (neutrophil)-positive cells in pancreatic tissues compared with that of the cerulein group (Fig. 4A, B). Flow cytometry assay showed an increased population of neutrophils in blood, and macrophages and neutrophils in pancreas (Fig. 4C, D). As previously reported [19], the expression levels of cytokines, including Cxcl10 and Mcp1, Il6, Il1β, and Tnfα, were significantly induced by stimulation with cerulein, and J14 further accelerated the activation of macrophages and neutrophils in the pancreas (Fig. S4A, B).

Next, we isolated BMDMs that were then stimulated by MCSF for 7 days to differentiate into M1 phenotype macrophages and cocultured them with the isolated acini (Fig. 4E). LPS was served as a positive control. Coincubation of M1 macrophages with cerulein-stimulated acini led to a significant elevation of IL6 and TNFα secretion (Fig. 4F). After coincubation with cerulein-stimulated acini transfected with Srxn1 siRNA, IL6 and TNFα release was substantially increased (Fig. 4F). Macrophages can actively phagocytose trypsinogen and activate it to induce an inflammatory response. We observed that more amylase accumulated in macrophages when cocultured with acini transfected with Srxn1 siRNA than that of the control (Fig. 4G). Next, we isolated neutrophils from marrow and added them to adherent acinar cells (Fig. 4H and Fig. S4C). Likewise, either J14 or Srxn1 siRNA significantly increased the RNA expression of Il6, Cxcl10, and Tnfα in neutrophils (Fig. 4I and Fig. S4D). Thus, these data indicated that inhibition of Srxn1 promoted the accumulation and activation of M1 phenotype macrophages and neutrophils, inducing a more vigorous inflammatory response in AP.

### ER stress-mediated activation of cathepsin B is responsible for the Srxn1 inhibition-mediated effects

**Fig. 5:**
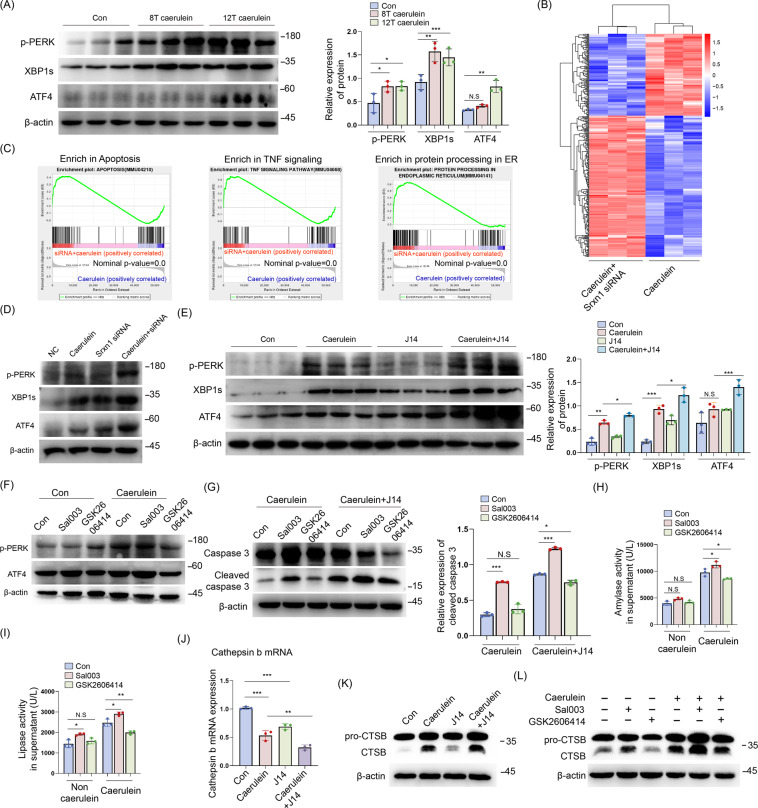
ER stress-mediated activation of cathepsin B is responsible for the Srxn1 inhibition-mediated effects. **A** Administration of cerulein via 8-hourly or 12-hourly injections led to significantly increased expression of p-PERK, XBP1s, and ATF4 in pancreatic tissues. **B** The transcriptomic profiles of acinar cells with Srxn1 knockdown or control stimulated by cerulein (50 nM) for 6 h. **C** GSEA showed that siRNA led to the enrichment of apoptosis, TNF, and protein-processing signaling pathways in acinar cells. **D** siRNA led to markedly increased expression of ER stress markers in acinar cells. **E** J14 significantly induced ER stress upon stimulation with cerulein compared to cerulein alone in mice. **F** Sal003 induces ATF4 expression, and GSK2606414 inhibits p-PERK and ATF4 expression. **G** Activation of ER stress by Sal003 facilitated apoptosis, while inhibition of ER stress by GSK2606414 inhibited apoptosis in acinar cells. In the presence of J14, inhibition of ER stress alleviated apoptosis nearly to the level of the cerulein group. **H**, **I** Sal003 increased trypsin activity, while GSK2606414 decreased trypsin activity in acinar cells. **J** Cathepsin B RNA expression was decreased by cerulein and Srxn1 siRNA. **K** Cerulein activated CTSB, which could be further enhanced by J14. **L** Inhibition of ER stress alleviated the activation of CTSB, while inducing ER stress markedly promoted the activation of CSTB in acinar cells. Con control; *p < 0.05, **p < 0.01, ***p < 0.001. Data represent three or more experiments for in vitro assays and eight for in vivo assays.

The ER stress markers, p-PERK, XBP1s, and ATF4, were significantly upregulated in AP (Fig. 5A). The transcriptional profiles of isolated acini transfected with Srxn1 siRNA or control were determined. In total, 357 differentially expressed genes were identified by a cutoff value of *p* < 0.05 and log_2_ fold change >1 or <−1 (Fig. 5B). Consistent with the aforementioned results, gene set enrichment analysis (GSEA) showed that knockdown of Srxn1 resulted in enrichment in apoptosis and the TNF signaling pathway in acinar cells (Fig. 5C). Interestingly, the knockdown of Srxn1 also resulted in enrichment in protein processing in the ER (Fig. 5C). Then, we confirmed the stronger induction of ER stress in the Srxn1- knockdown group than in the control group (Fig. 5D). This result was reinforced by the finding that J14 led to increased expression of p-PERK, XBP1s, and ATF4 compared with the control upon stimulation with cerulein in vivo (Fig. 5E). Sal003 and GSK2606414 are specific activators or inhibitors of ER stress, respectively. In isolated acinar cells, Sal003 increased the expression of ATF4, indicating the activation of ER stress, and GSK2606414 showed the opposite effect (Fig. 5F). The expression of cleaved caspase 3 was induced by Sal003 but reduced by GSK2606414 upon cerulein treatment, and importantly, GSK2606414 reduced its expression in the cerulein + J14 group near the level of the cerulein group (Fig. 5G). In addition, activation of ER stress increased trypsin activity, while inhibition of ER stress decreased trypsin activity in the presence of cerulein (Fig. 5H, I). This evidence suggests that ER stress contributes to the proapoptotic and protrypsin activity induced by Srxn1 inhibition in AP.

Under pathological conditions, trypsinogen is converted to active trypsin by the lysosomal hydrolase cathepsin B (CTSB) in acinar and macrophage cells. We speculated that ER stress might influence the expression of CTSB, resulting in the activation of trypsin. Interestingly, either cerulein or J14 decreased the RNA expression of CTSB in acinar cells (Fig. 5J). However, regarding protein expression, cerulein induced the expression of the active form of CTSB and further increased it in the presence of J14 (Fig. 5K). Activation of ER stress by Sal003 positively regulated the expression of the active form of CTSB (Fig. 5L). In contrast, inhibition of ER stress by GSK2606414 decreased its active form in acinar cells (Fig. 5L). Thus, the activity of CTSB was regulated by ER stress in acinar cells. In summary, these data indicated that ER stress-induced activation of CTSB was responsible for the Srxn1 inhibition-mediated effects in AP.

### AAV-mediated overexpression of Srxn1 protects against AP

**Fig. 6:**
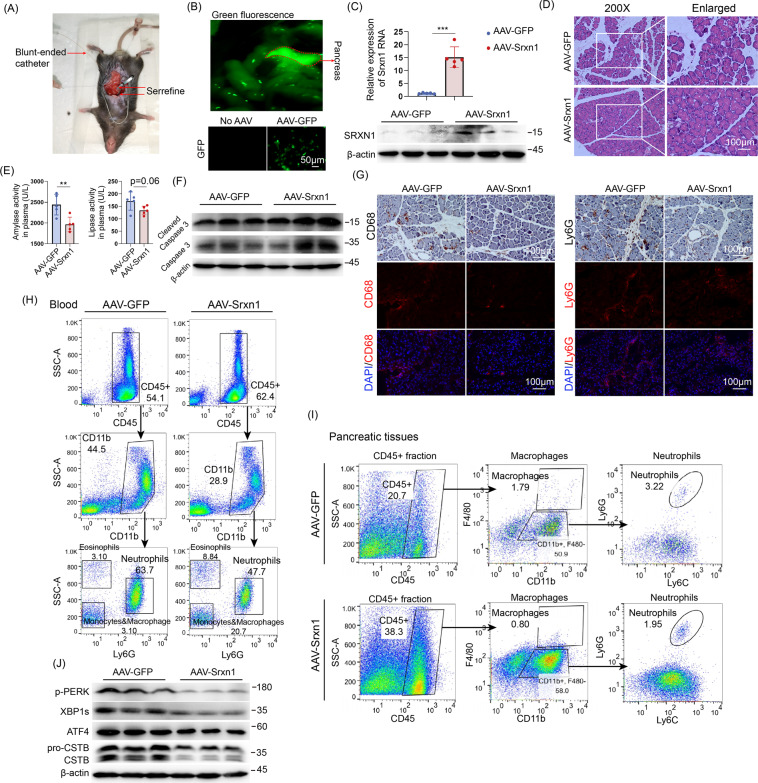
AAV-mediated overexpression of Srxn1 protects against AP. **A** Schematic of the microinfusion system for intraductal administration of AAV. **B** Fluorescence imaging of pancreas (upper) and pancreatic sections (lower) after AAV administration for 14 days. **C** SRXN1 RNA and protein was upregulated by AAV-Srxn1 in pancreatic tissues. **D** AAV-Srxn1 markedly reduced amylase and lipase activity in plasma compared with control. **F** Cleaved caspase 3 was decreased in AAV-Srxn1 group. **G** IHC and IF staining of CD68 and Ly6G showed a reduced infiltration of M1 macrophages and neutrophils by AAV-Srxn1 in pancreas. **H** Flow cytometry analysis of blood showed a decrease of neutrophils (CD45^+^/CD11b^+^/Ly6G^+^) in AAV-Srxn1 group. **I** Flow cytometry analysis of pancreatic tissues showed a decrease of infiltrating neutrophils (CD45^+^/CD11b^+^/Ly6C^+^/Ly6G^+^) and macrophages (CD45^+^/CD11b^+^/F4/80^+^) in AAV-Srxn1 group. **J** AAV-Srxn1 decreased the expression of p-PERK, XBP1s, and the active form of CSTB in the pancreas. **p < 0.01, ***p < 0.001. *n* = 5.

To examine that whether overexpression of Srxn1 would be beneficial for AP in vivo, we used AAV vector (AAV9-CMV-Srxn1-GFP, AAV-Srxn1) to introduce Srxn1 by intraductal administration (Fig. 6A). Visualized pancreas and green fluorescence in the histological section suggested a high efficiency of AAV system in pancreas (Fig. 6B), and Srxn1 was markedly overexpressed (Fig. 6C). When induced by cerulein, overexpression of Srxn1 reduced the histological severity and the trypsin activities in plasma compared with control (Fig. 6D, E). Cleaved caspase 3 was less expressed in pancreas with the administration of AAV-Srxn1, suggesting that Srxn1 attenuated apoptosis during AP (Fig. 6F). IHC and IF staining of CD68 and Ly6G showed that overexpression of Srxn1 attenuated the infiltration of M1 macrophages and neutrophils in pancreas (Fig. 6G). Furthermore, flow cytometry was used to examine the ratios of myeloid cell populations in blood and pancreas, and comparing with control, reduced ratios of neutrophils in blood and reduced ratios of neutrophils and macrophages were observed in mouse with AAV-Srxn1 (Fig. 6H). Furthermore, Srxn1 suppressed the ER stress and activation of CSTB in pancreas when stimulated by cerulein (Fig. 6I). Thus, these data demonstrated that overexpression of Srxn1 by intraductal administration of AAV vector protected against AP in mice.

## Discussion

Pancreatic toxins could provoke the fusion of zymogen granules with lysosomes to activate trypsinogen to trypsin, causing apoptosis [20, 21]. Damage-associated molecular patterns and other components stimulate and activate inflammation [22]. Infiltrating macrophages can phagocytose trypsinogen to exacerbate acute inflammation locally by converting trypsinogen to trypsin [23]. Thus, pathological acinar cells and an activated inflammatory response are the core processes of AP. In our study, we investigated the role of Srxn1 in AP by surveying its function and the underlying mechanisms of regulating ROS, apoptosis, and the inflammatory response in isolated acini and mice.

Acinar cells possess a fine-equipped biosystem to maintain the balance between ROS generation and endogenous scavenging [5]. In various models and clinical investigations, oxidative stress has been firmly demonstrated and associated with the severity of AP [24]. Oxidative stress contributes to the inflammatory cascade, resulting in the activation of macrophages and neutrophils and the production of proinflammatory cytokines, such as IL6, IL1β, and TNFα [25]. Thus, ROS play an important role in AP. Overexpression of thioredoxin-1, a critical protein for intracellular maintenance of the redox balance, largely ameliorated the severity and inflammation of experimental AP [26]. Previous works indicated that Srxn1 protects cardiac progenitor cells against oxidative stress-induced apoptosis by decreasing ROS generation and increasing primary antioxidant systems [12]. In our research, we verified that inhibition of Srxn1 facilitated ROS generation and induced ER stress, which promoted the release of trypsin, apoptosis, and the activation of M1 macrophages and neutrophils in AP.

Macrophages and neutrophils are the main inflammatory cells infiltrating the pancreas during AP [20]. Inflammatory cells are further activated by acinar cellular contents and amplify the production of proinflammatory cytokines, including TNFα, IL1β, IL6, and IL18 [27, 28]. The extent of acinar injury determines the number of recruited inflammatory cells and the degree of activation. We found that Srxn1 controlled the ROS induced by cerulein in the pancreas, and inhibition of Srxn1 strongly augmented the inflammatory response by activating M1 macrophages and neutrophils. Macrophages can phagocytose large amounts of zymogens when disposing of injured acinar cells or released cellular components [23]. We observed an enhanced absorption of amylase in macrophages cocultured with acinar cells when Srxn1 was inhibited, emphasizing its role in controlling the activation of trypsin during AP.

The balance between protein production and recycling of unwanted proteins is easily disrupted upon AP in acinar cells [29]. During AP, especially SAP, the UPR eventually activates apoptosis. ER stress can directly initiate inflammatory pathways through the PERK-eIF2α signaling pathway, thereby reducing protein synthesis and increasing the ratio of NF-κB to IκB [30]. We confirmed that ER stress is activated in cerulein-induced AP and that inhibition of Srxn1 further augmented ER stress-mediated apoptosis and the inflammatory response in acinar cells. Intracellular or extracellular sources of ROS destroy Ca^2+^ restoration in the ER lumen, leading to loss of Ca^2+^ oscillations in the cytosol and Ca^2+^ overload in mitochondria [20]. Thus, Srxn1 inhibition-mediated augmentation of apoptosis and inflammation is induced by ROS-triggered ER stress in acinar cells and mice. Furthermore, ER stress might possess a reciprocal regulation of the activity of trypsin in AP. Cathepsin B is responsible for the conversion of trypsinogen to active trypsin in acinar cells. During AP, cathepsin B expression and activity are increased [31]. Inhibition of ER stress by tauroursodeoxycholic acid reduced cathepsin B activity in rat acini [14]. Our results first showed that activation of ER stress increased the activity of cathepsin B, while inhibition of ER stress decreased it in isolated acinar cells. Therefore, we concluded that the ROS/ER stress/cathepsin B axis is the underlying mechanism of Srxn1-mediated regulation of trypsin activation, apoptosis, and activation of macrophages and neutrophils in AP.

Furthermore, we demonstrated that intraductal administration of AAV carrying Srxn1 could ameliorate trypsin activation, apoptosis, and the inflammatory response in mice. These results provide a potential therapeutic strategy for AP.

## Materials and methods

### Animal models

C57BL/6J mice were purchased from HUNAN SJA Laboratory Animal Co., Ltd. Mice were housed under pathogen-free conditions. The project was approved by The Second Xiangya Hospital, Central South University, and complied with the animal care and use guidelines. No randomization and no blinding was used for animal study.

For the mild form of AP, male mice (6−8 weeks old) were fasted for 12 h and intraperitoneally injected with cerulein (50 µg/kg body weight) at 1-h intervals 4, 8, and 12 times. One severe form of AP was induced in C57BL/6J mice by partial duct ligation of the pancreatic duct, followed by three doses of cerulein (50 µg/kg body weight) at 1-h intervals after surgery, which was modified from previous reports [19]. The animals were sacrificed 3 days after the first injection. Another severe AP model was induced by 3-hourly intraperitoneal injections of 3.3 g/kg L-arginine, and the animals were sacrificed 3 days after the first injection [32]. The animals with jaundice were excluded for further evaluation.

For the pharmacological inhibition of SRXN1, mice were intraperitoneally injected with J14 (in 10% DMAC + 10% Tween 80 + 80% saline) at a dose of 50 mg/kg body weight once a day for 2 weeks before induction of AP.

For knockdown of Srxn1 expression, C57BL/6J mice were intravenously treated with Srxn1 siRNA or negative control (20 nmol/injection, provided by RiboBio, China) two times a week for 4 weeks. The targeted sequence of Srxn1 siRNA was GGCGACTACTACTATTCCT.

### Histological analysis

Pancreatic and liver tissues were collected and immediately fixed in 10% paraformaldehyde. The fixed tissues were sent to Servicebio (Wuhan, China) for paraffin embedding and cut into 4-μm sections. The severity of pancreatitis and the mean score of each sample were analyzed by two researchers according to a previous report.

### Analysis of trypsin activity

The trypsin and lipase activities in plasma and cell supernatant were measured by a clinical analysis system. Amylase activity was determined by a kit based on the EPS substrate method and measured by an AU1000 system (Beckman, America). Lipase activity was determined by a kit based on the methyl triazine substrate method and measured by the same system.

### Acinar cell preparation

The protocol for the isolation of acinar cells from mice was modified from a previous report [33]. Briefly, mice were killed by CO_2_ asphyxiation, and the complete pancreas was collected. Collagenase IV solution (HBSS 1× containing 10 mM HEPES, 200 U/ml collagenase IV, and 0.25 mg/ml trypsin inhibitor) was added to digest the pancreas for 40 min on a shaker (80 rpm/min). Then, stop solution (HBSS 1× containing 5% FBS and 10 mM HEPES) was added to stop the enzymatic reaction. The sample was centrifuged for 3 min at 250 × *g*, resuspended with 10 ml of complete medium (Waymouth’s medium containing 2.5% FBS, 1% pen/strep, 0.25 mg/ml of trypsin inhibitor, and 25 ng/ml of recombinant human epidermal growth factor), passed through a 100-μm filter, and collected the isolated acini. The acini were cultured with complete medium at 37 °C under a 5% (v/v) CO_2_ atmosphere.

### BM-derived macrophage preparation

The femur and tibia of C57BL/6J mice were cut under sterile conditions. BM was washed out with sterile DMEM three times. The medium was passed through a cell 70-μm strainer, washed two times with sterile PBS, counted, and seeded in six-well plates or chamber slides for immunofluorescence staining with DMEM containing 10% FBS, 1% pen/strep, and 20 Ng/ml MCSF. Six hours later, the nonattached cells were removed. Cells were ready to use after a culture of 7 days.

For coculture of macrophages with acini, acinar cells were isolated and transfected with siRNA or negative control for 2 days or treated with J14 (5 μM) for 6 h. Then, acinar cells were carefully collected and centrifuged for 3 min at 250 × *g* and stimulated with cerulein (100 nM). Acinar cells were added to six-well plates or chamber slides and cultured for 6 h. The supernatant was harvested and centrifuged at 12000 × *g* for 10 mins. Cells were washed two times with sterile PBS. Cells were fixed with 4% paraformaldehyde when prepared for immunofluorescence staining. For PCR, TRIzol (Invitrogen, America) was added, followed by RNA extraction.

### Isolation of BM-derived neutrophils

BM-derived neutrophils were prepared from C57BL/6J mice (6−8 weeks old) according to an established protocol [34]. Briefly, femur and tibia bones were flushed with RPMI 1640, and cells were collected and treated with ACK lysis buffer (A1049201, Fisher Scientific, America) to lyse red blood cells. The samples were centrifuged at 1600 rpm/min. The cell pellet was suspended in HBSS with phenol red, and neutrophils were separated and collected by Percoll density-gradient centrifugation. Giemsa staining was used to identify neutrophils. The neutrophils were incubated with RPMI 1640 containing 10% FBS and 1% pen/strep.

For coculture of neutrophils with acini, acinar cells were isolated and transfected with siRNA or negative control for 2 days or treated with J14 (5 μM) for 6 h. Then, neutrophils were added for coincubation for 6 h. The supernatant containing neutrophils was collected and centrifuged at 1600 rpm/min for 5 min, and the cell pellet was collected. The supernatant was centrifuged at 12000 × *g* for 10 min. TRIzol was added to the cell pellet, followed by RNA extraction.

### Immunofluorescence staining

Immunofluorescence staining was performed on 4-μm paraffin sections or chamber slides. The sections were deparaffinized and incubated with 3% H_2_O_2_ in the dark for 15 min, followed by epitope retrieval with sodium citrate buffer (10 mM sodium citrate and 0.05% Tween 20 at pH 6.0) at 96 °C for 30 min. Then, the sections or chamber slides were blocked with 5% goat serum (16210064, Fisher Scientific, America). Anti-CD68 (14068182, eBioscience, America) was used as a marker for M1 macrophages, anti-Ly6G (14593182, eBioscience, America) for neutrophils, and anti-amylase (sc-46657, Santa Cruz, America) for acinar cells. Nuclei were stained with DAPI-containing antifade reagent (P36935, Invitrogen, America). Images were acquired using an Olympus fluorescence microscope or a Leica confocal microscope.

### Protein extraction and Western blotting

Tissues or cells were collected and lysed in RIPA buffer mixed with protease inhibitors (TargetMol, America) and incubated on ice for 30 min. The lysates were centrifuged at 14000 g for 10 min at 4 °C, and the supernatant was collected. The denatured proteins were added to the chamber for electrophoresis conducted for the proper length of time, followed by transfer onto PVDF membranes. The membranes were blocked in 3% BSA for 1 h at room temperature. Antibodies against SRXN1 (ab92298, Abcam, America), cleaved caspase 3 (9661, CST, America), β-actin (AC038, Abclonal, China), p-PERK (AP0886, Abclonal, China), XBP1s (A1731, Abclonal, China), ATF4 (A0201, Abclonal, China), and Cathepsin B (31718, CST, America) were used. The next day, the primary antibody was washed away with TBST solution, and the secondary antibody (Abclonal, China) was added for 1 h at room temperature. Finally, the immune complexes were detected via enhanced chemiluminescence (Life Tec, America). Quantification of the bands was carried out with ImageJ software (Version 11).

### RNA extraction and quantitative real-time PCR

Tissues and cells were treated with TRIzol reagent (Invitrogen, America) for 10 min, followed by centrifugation at 12000 g for 15 min at 4 °C. Then, the RNA to be suppressed was collected and mixed with isopropanol for isolation of the RNA. After the RNA was obtained, RNA purity and concentration were analyzed using a Nanodrop 1000 spectrophotometer (Thermo Fisher, America). cDNA was synthesized using a high-capacity cDNA reverse transcription kit (Life Tec, America). The primers were listed as follows: Srxn1, 5′-GTGCACAACGTACCAATCG-3′ (forward) and 5′-GCCCCCAAAGGAATAGTAGTAG-3′ (reverse); Il6, 5′-CTCCCAACAGACCTGTCTATAC-3′ (forward) and 5′- CCATTGCACAACTCTTTTCTCA-3′ (reverse); Il1β, 5′-TCGCAGCAGCACATCAACAAGAG-3′ (forward) and 5′- TGCTCATGTCCTCATCCTGGAAGG-3′ (reverse); Tnfα, 5′-ATGTCTCAGCCTCTTCTCATTC-3′ (forward) and 5′- GCTTGTCACTCGAATTTTGAGA-3′ (reverse); Cxcl10, 5′-CAACTGCATCCATATCGATGAC-3′ (forward) and 5′- GATTCCGGATTCAGACATCTCT-3′ (reverse); Mcp1, 5′-TTTTTGTCACCAAGCTCAAGAG-3′ (forward) and 5′- TTCTGATCTCATTTGGTTCCGA-3′ (reverse); Cathepsin B, 5′-CTCATGTAGGCTGCTTACCATA-3′ (forward) and 5′- TCTCCTTCACACTGTTAGACAC-3′ (reverse). qRT-PCR was performed with 2X Universal SYBR Green Fast qPCR mix (Abclonal, China) on a LightCycler 96 system (Roche, America).

### Transferase-mediated d-UTP nick-end-labeling (TUNEL) assay

A TUNEL kit was purchased from Beyotime, China, and was used according to the guidelines. Briefly, the sections were deparaffinized by xylene two times and then dehydrated by ethyl alcohol. Protease K was added to digest proteins. TUNEL solution was added and incubated for 60 min in the dark, the sections were washed with PBS three times, and antifade reagent was added. Images were acquired using an Olympus fluorescence microscope.

### Cytokine measurement in supernatant/serum

An enzyme-linked immunosorbent assay was performed to measure TNFα (Abclonal, China) and IL6 (Abclonal, China) in serum and cell culture supernatant according to the manufacturer’s instructions.

### Biochemical analysis

For malondialdehyde (MDA), superoxide dismutase (SOD), and GSH (glutathione)/GSSH (oxidized glutathione disulfide) activity, kits were purchased from Beyotime (China) and performed according to the manufacturer’s instructions. TBA reagent was added to each vial to generate the MDA-TBA adduct, and the absorbance was measured on a microplate reader (Fisher Scientific, America) at 532 nm for the colorimetric assay. For SOD activity, prepared WST-8/enzyme mix and reaction solution were added to each sample and incubated for 30 min, and the absorbance was measured on a microplate reader (Fisher Scientific, America) at 450 nm. For GSH/GSSH activity, prepared DTNB/glutathione reductase mix and NADPH solution were added and incubated for 25 min, and the absorbance was measured on a microplate reader (Fisher Scientific, America) at OD 412 nm. The result was calculated based on the GSH and GSSH standard curves.

### siRNA transfection

siRNA was designed by the siCatch^TM^ siRNA design system and synthesized by RiboBio (China). Transfection (100 pmol/ml) was performed using Lipofectamine^TM^ RNAiMAX reagent. The efficiency was verified by determining Srxn1 mRNA and protein expression.

### SRXN1 lentivirus

For overexpression of Srxn1 in vitro, lentivirus vectors harboring full-length Srxn1 were designed, and a scrambled shRNA was used as the negative control. The lentivirus vectors were purchased from GeneChem Co., Ltd. (China). The multiplicity of infection was 20, and the period was 4 days. The efficiency was verified by qRT-PCR and Western blots.

### Delivery AAV to pancreas by intraductal administration

AAV administration was referred to a previous report. [35] Briefly, prepare 150 μL per mouse of 3 × 10^12^ vector genomes of AAV in PBS. Anesthetize mice, incise the abdomen at the midline, and expose the pancreatic duct. Place serrefines on the common bile duct to prevent infusion into the liver or back into the duodenum. Set up the infusion equipment, connect the back end of the catheter to the infusion needle, and start infusion at 6 μL per min.

### Flow cytometry analysis

Collect the blood, pipette 10 mL of ACK buffer to mix with the blood and leave for 10 min, and centrifuge the cells. Add cold PBS to each tube, resuspend, count the cells and transfer 1 × 10^6^ cells into a new EP tube.

Isolation of pancreas leukocytes was performed according to an established protocol. [36] Briefly, cut the pancreas into 1−2-mm pieces, add 0.4 mg/mL collagenase P (Roche), and incubate the pancreas for 30 min at 37 °C. Then, stain the pancreas through a 70-μm, and a 40-μm cell strainer, centrifuge at 300 × *g*, and resuspend, count the cells. Transfer 1 × 10^6^ cells into a new EP tube.

Centrifuge for 4 min, add 100 μL of cold PBS to resuspend the cells, and add 100 μL of antibodies mix (viability staining 565388, CD11b 550993, Ly6G 551461, F4/80 565411, CD45 563891, and Ly6C 553104) for 45 min at room temperature. All antibodies were purchased from BD Pharmingen. The cells were then fixed and analyzed by a flow cytometer (BD Biosciences). Data analysis is carried out using FlowJo (Version 11) software program.

### Statistical analysis

All data are expressed as the mean ± standard error using GraphPad Prism 8. Animal experiments were performed at least five or eight times, and cell experiments were performed at least three times. Unpaired Student’s *t* test was used to evaluate the significance between two groups (using parametric test when justified as normal distribution and homoscedasticity otherwise, using nonparametric test), and one-way analysis of variance was used to determine the differences among multiple groups. A value of *P* < 0.05 was regarded as statistically significant.

## Supplementary information

Supplementary Figure legends

Supplementary Figure 1

Supplementary Figure 2

Supplementary Figure 3

Supplementary Figure 4
