# Supplementary material for: Sulfiredoxin-1 attenuates injury and inflammation in acute pancreatitis through the ROS/ER stress/Cathepsin B axis
Source: Cell Death Dis. 2021 Jun 17;12(7):626. doi: 10.1038/s41419-021-03923-1 (PMC8211864; doi:10.1038/s41419-021-03923-1)
Supplement: Supplementary file 1 — Supplementary Figure legends [file 41419_2021_3923_MOESM1_ESM.docx]

**Supplementary Figure 1**

(A) We performed partial duct ligation and injection of caerulein to establish the SAP model. Methylene blue was injected to mark the pancreatic duct after clamping the choledoch. The white arrow indicates the biliopancreatic duct, and the red arrow indicates the pancreatic duct. (B-C) The RNA expression of Sel1 and Btg2 was determined by qRT-PCR in the caerulein-induced AP models. Con, control; *p<0.05, **p<0.01, ***p<0.001. Data are from 8 experiments.

**Supplementary Figure 2**

(A) J14 (5 μM) decreased the DCF signal induced by H_2_O_2_ (100 nM) by a concentration dependent way in acinar cells. (B) Administration of Srxn1 siRNA increased amylase and lipase activity in plasm in AP. Con, control; NC, negative control; **p<0.01, ***p<0.001. Data are from 3 or more experiments.

**Supplementary Figure 3**

(A) J14 (5 μM) increased the DCF signal in acinar cells, and this signal became more obvious when stimulated by caerulein (100 nM). (B) The DCF signal was elevated in acinar cells transfected with Srxn1 siRNA compared to that in the control group. (C) Immunofluorescence staining of cleaved caspase 3 showed that J14 (5 μM) promoted apoptosis compared to the control upon stimulation with caerulein. Con, control.

**Supplementary Figure 4**

(A) Inhibition of Srxn1 significantly increased the RNA expression of proinflammatory cytokines, including Cxcl10, Mcp1, Il6, Il1β, and Tnfα, in pancreatic tissues. (B) Neutrophils were isolated from BM and verified by Giemsa staining. (D) Adding acini treated with J14 significantly increased the RNA expression of Tnfα, Il6, and Cxcl10 in neutrophils compared to adding normal acini. Con, control; *p<0.05, ***p<0.001. Data are from 3 experiments.
