# Supplementary figures and images for: Sulfiredoxin-1 attenuates injury and inflammation in acute pancreatitis through the ROS/ER stress/Cathepsin B axis
Source: Cell Death Dis. 2021 Jun 17;12(7):626. doi: 10.1038/s41419-021-03923-1 (PMC8211864; doi:10.1038/s41419-021-03923-1)

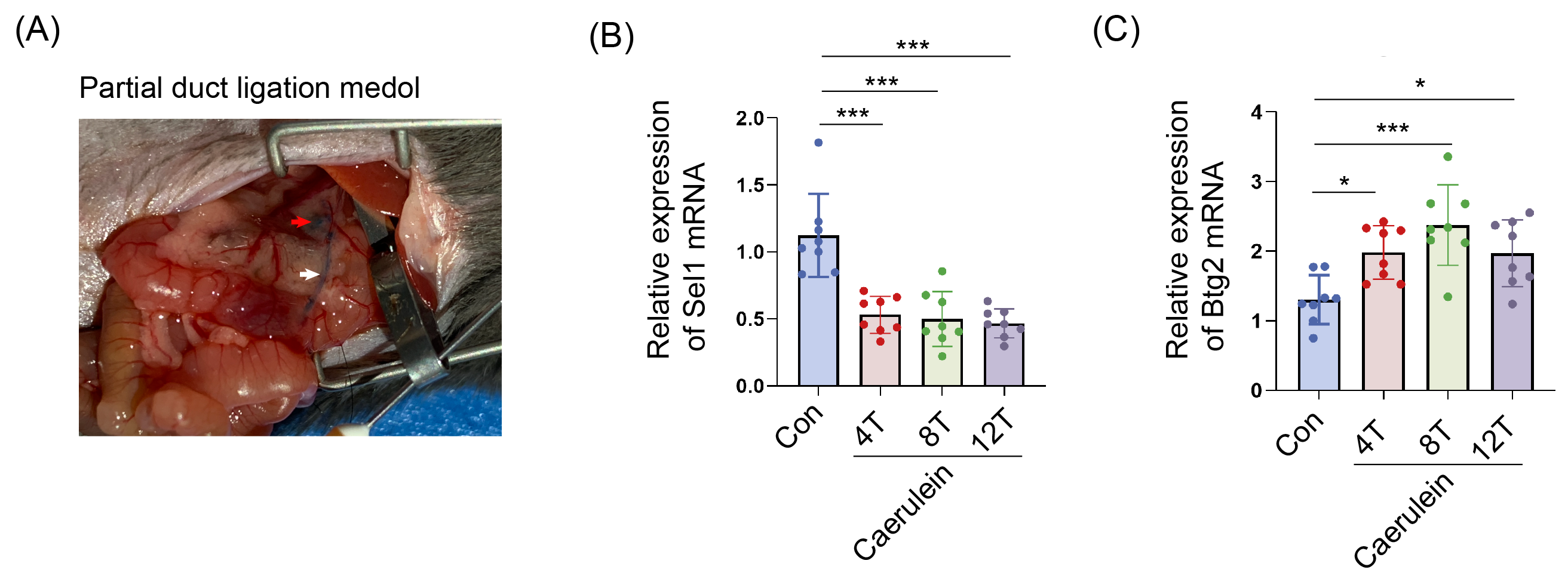

Supplement: Supplementary file 2 — Supplementary Figure 1 [file 41419_2021_3923_MOESM2_ESM.tif]

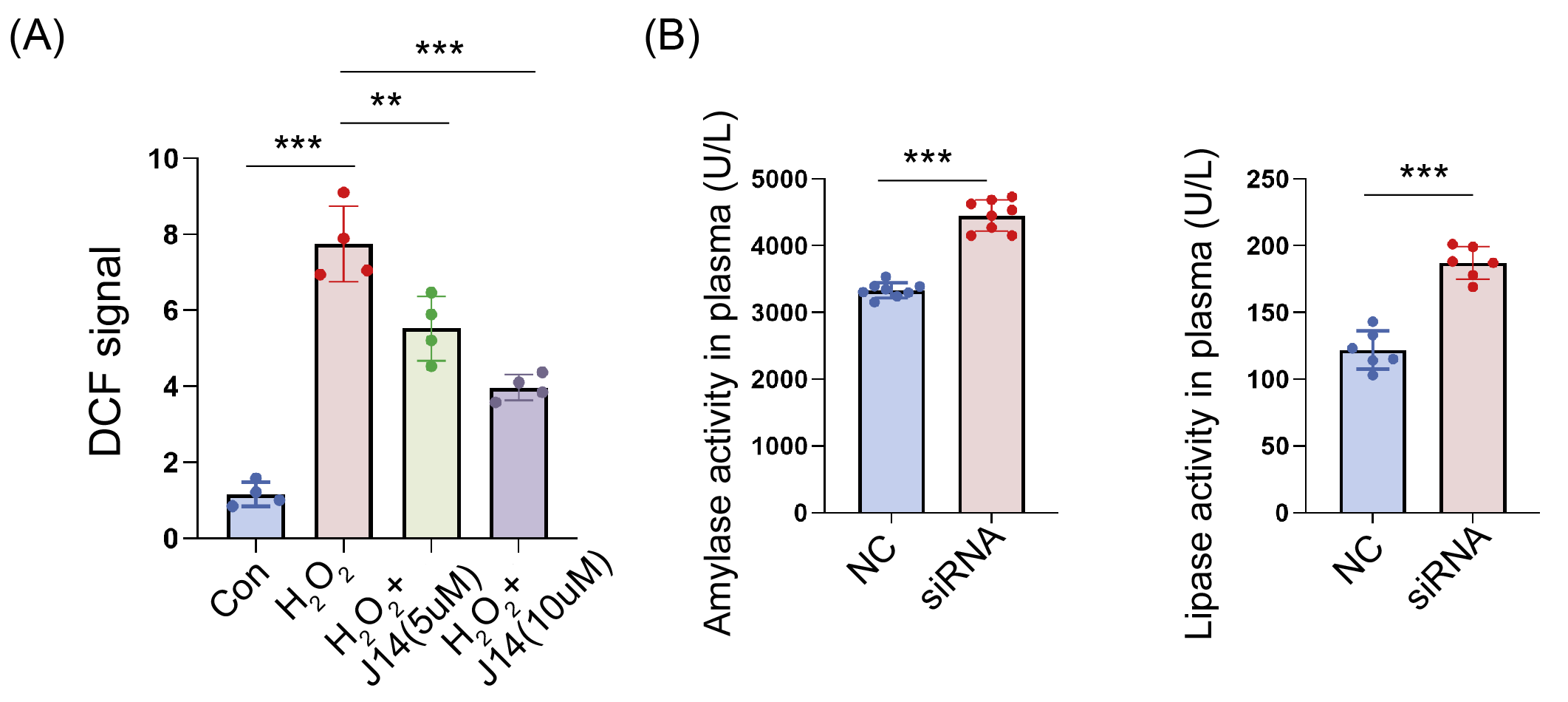

Supplement: Supplementary file 3 — Supplementary Figure 2 [file 41419_2021_3923_MOESM3_ESM.tif]

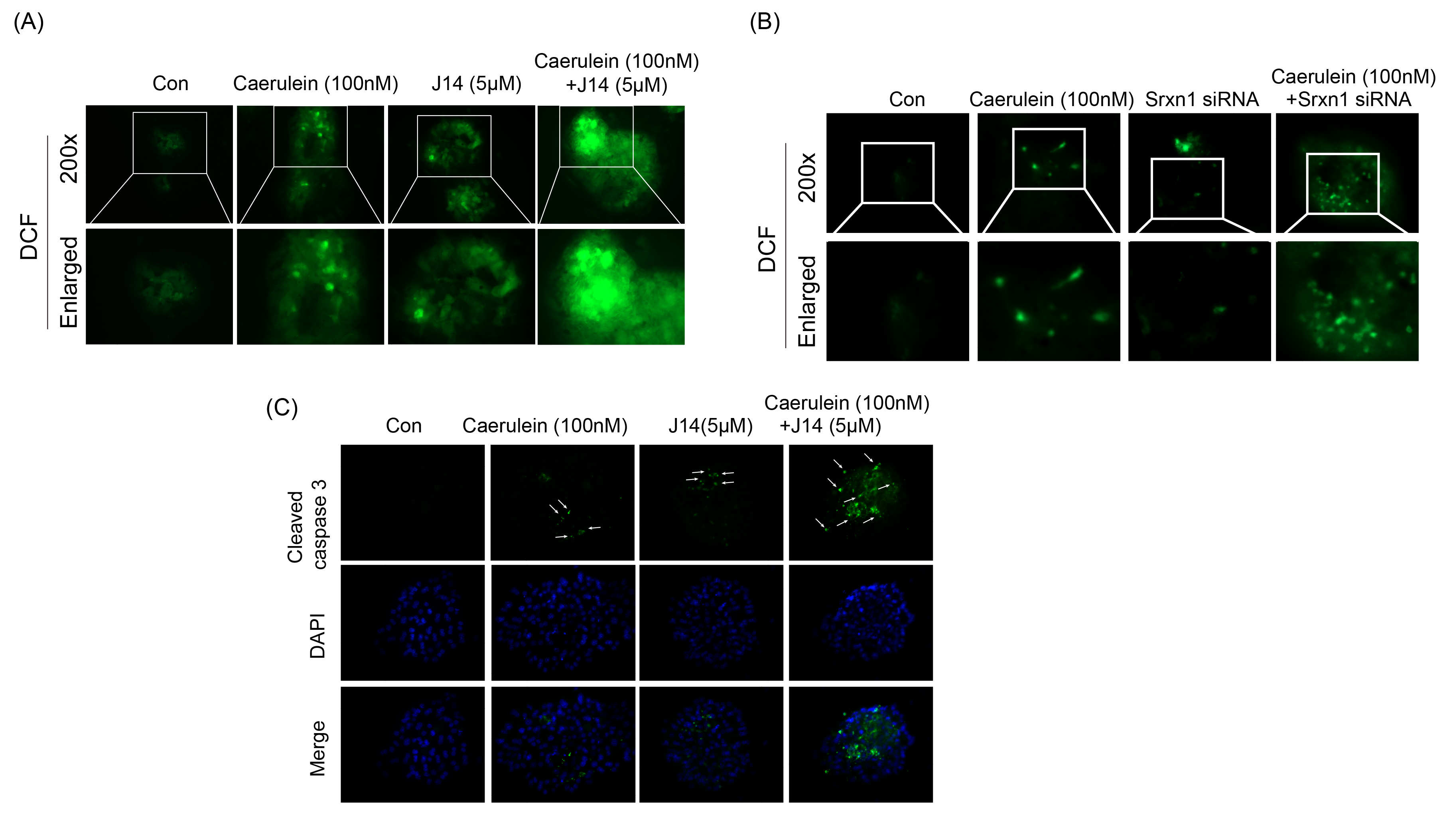

Supplement: Supplementary file 4 — Supplementary Figure 3 [file 41419_2021_3923_MOESM4_ESM.tif]

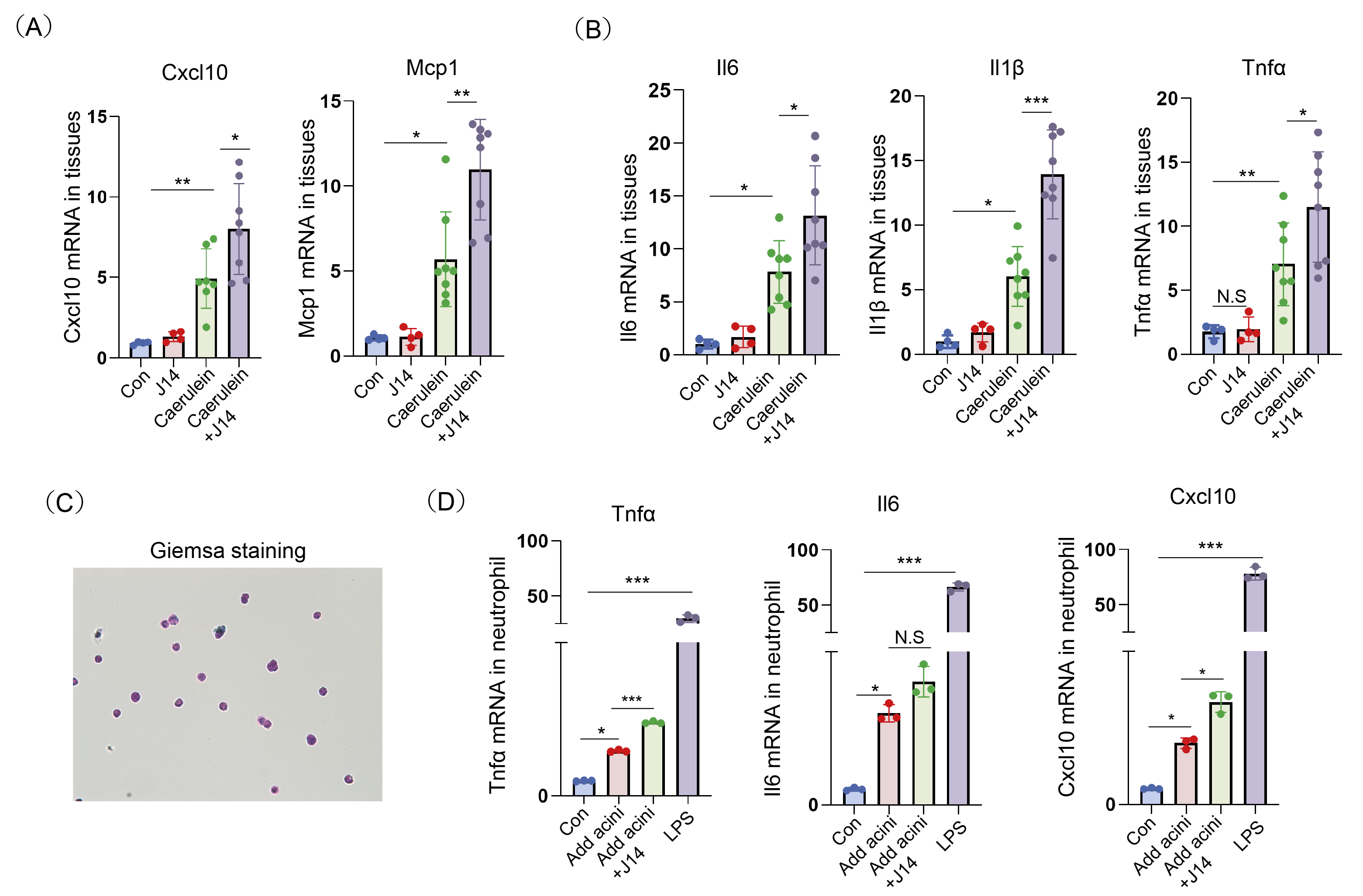

Supplement: Supplementary file 5 — Supplementary Figure 4 [file 41419_2021_3923_MOESM5_ESM.tif]
